# Supplementary material for: Differential gene expression analysis of spatial transcriptomic experiments using spatial mixed models
Source: Sci Rep. 2024 May 14;14:10967. doi: 10.1038/s41598-024-61758-0 (PMC11094014; doi:10.1038/s41598-024-61758-0)
Supplement: Supplementary file 1 — Supplementary Information 1. [file 41598_2024_61758_MOESM1_ESM.pdf]

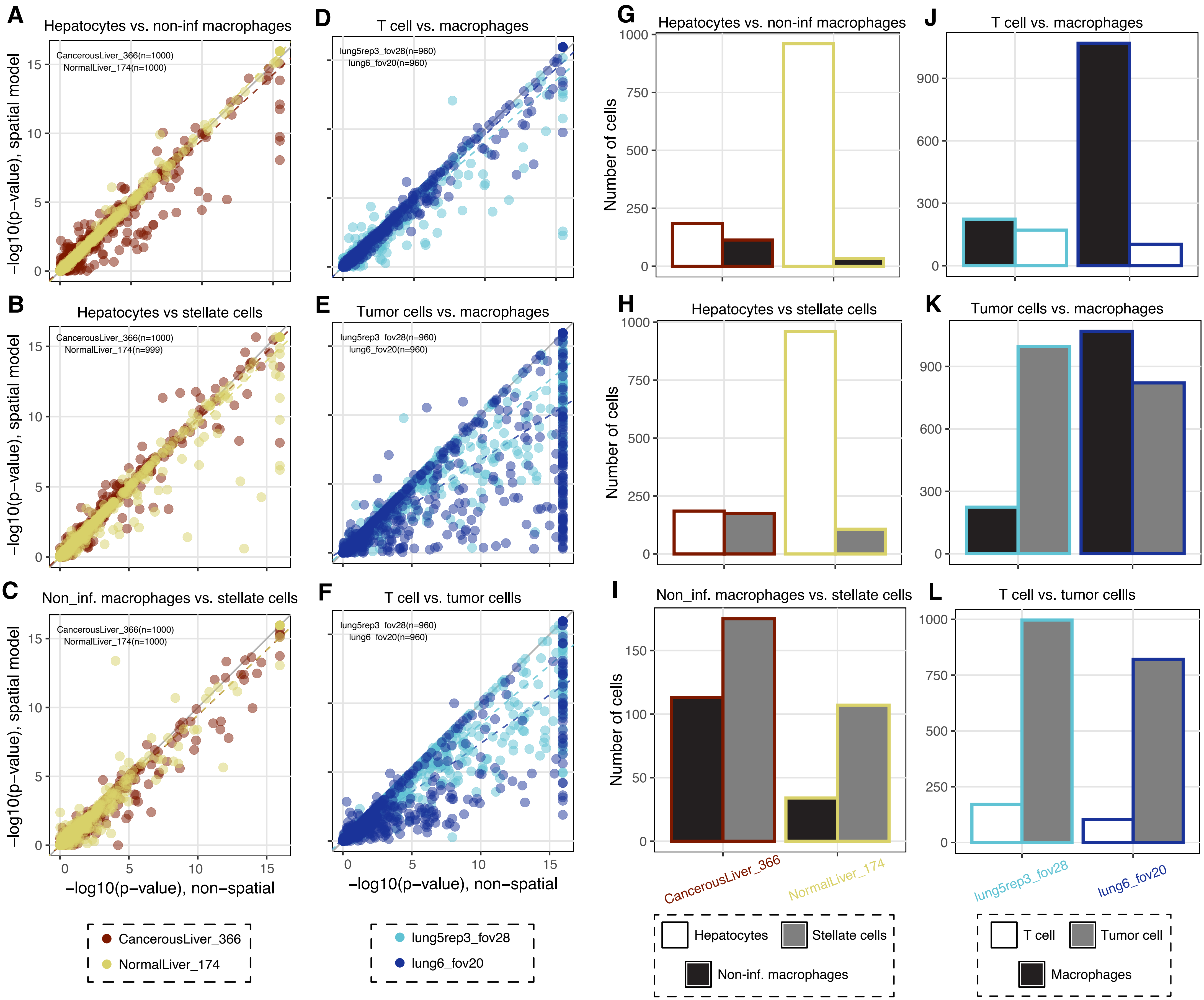

Supplementary Fig. S1. Cell type-level differential expression tests. Comparison between non-spatial and spatial (exponential model) differential expression tests. **A-F** Each point corresponds to the -log10(p-value) resulting from a (non-spatial or spatial) linear model fit between the expression of a gene and a binary variable indicating whether a cell belongs to either of the cell types. The p-values indicate if the gene is differentially expressed (model coefficient different to zero) for a specific biological annotation compared to the rest of the cells. The solid line indicates a 1:1 correspondence (i.e., non-spatial and spatial models yield the same p-values). The colored dashed lines indicate the linear trend of the p-values for each sample. If a colored line lies below the solid line, p-values from the non-spatial model tend to be larger than those from the spatial model. **G-L** Number of cells used in the differential expression tests. The effect of spatial tests in moderating excess of small p-values is larger when both cell types are abundant.
